# The manipulation of gene expression and the biosynthesis of Vitamin C, E and folate in light-and dark-germination of sweet corn seeds

**DOI:** 10.1038/s41598-017-07774-9

**Published:** 2017-08-08

**Authors:** Fengyuan Liu, Nan Xiang, Jian Guang Hu, Yan Shijuan, Lihua Xie, Charles Stephen Brennan, Wenjie Huang, Xinbo Guo

**Affiliations:** 1 School of Food Science and Engineering, South China University of Technology, Guangzhou, 510641 China; 2 Crop Research Institute, Guangdong Academy of Agricultural Sciences, Guangzhou, 510640 China; 3 Key Laboratory of Crops Genetics Improvement of Guangdong Province, Guangzhou, 510640 China; 4 Agro-Biological Gene Research Center, Guangdong Academy of Agricultural Sciences, Guangzhou, 510640 China; 5 Department of Wine, Food and Molecular Bioscience, Lincoln University, Canterbury, 7647 New Zealand

## Abstract

This study investigates the potential interrelationship between gene expression and biosynthesis of vitamin C, E and folate in sweet corn sprouts. Germination of sweet corn kernels was conducted in light and dark environments to determine if this relationship was regulated by photo-illumination. Results indicated that light and dark environments affected the *DHAR*, *TMT* and *GTPCH* expression and that these genes were the predominant genes of vitamin C, E and folate biosynthesis pathways respectively during the germination. Levels of vitamin C and folate increased during the germination of sweet corn seeds while vitamin E had a declining manner. Sweet corn sprouts had higher vitamin C and E levels as well as relevant gene expression levels in light environment while illumination had little influence on the folate contents and the gene expression levels during the germination. These results indicate that there might be a collaborative relationship between vitamin C and folate regulation during sweet corn seed germination, while an inhibitive regulation might exist between vitamin C and E.

## Introduction

Vitamins are essential nutrients for all living creatures, they play a critical role in diverse metabolisms of living organisms. Lack of certain vitamins has been shown to induce a number of diseases, therefore governments use bio fortification of nutrients (mainly vitamins in plants) as a mechanism to improve the nutritional value of foods^1^. Over the past decade, the processes of synthesis of vitamins in plants have been investigated extensively from a plant molecular biology view point, and it is possible to increase the levels of vitamins in specific plants via gene technology^2^. As more and more pathways are understood, a more complex metabolism network has been shown to exist which indicates that these pathways may have interdependent regulations on each other^3^.

**Figure 1:**
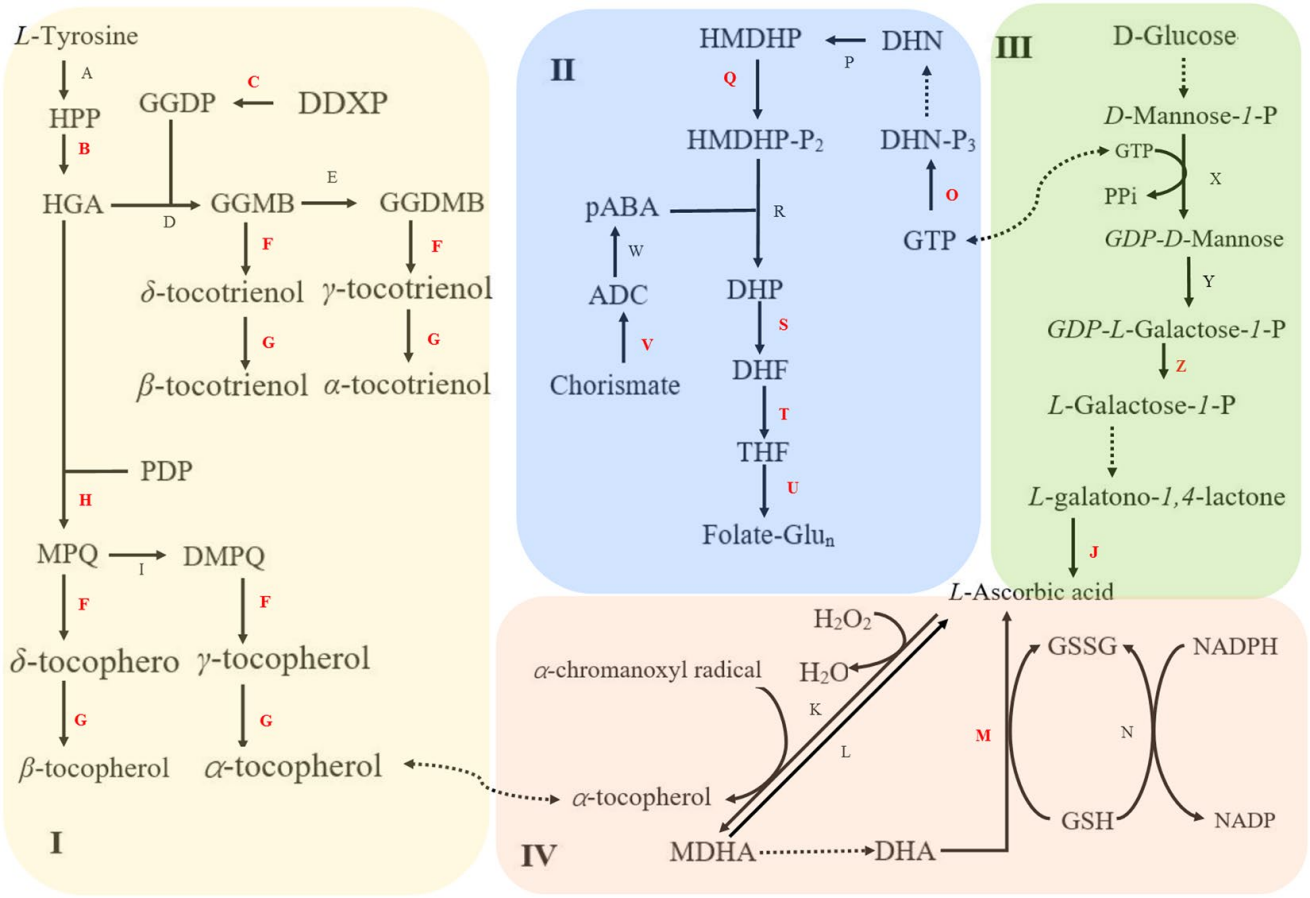
Pathways investigated in this study. (I) Vitamin E biosynthesis pathway. (II) Folate biosynthesis pathway. (III) Vitamin C biosynthesis pathway. (IV) Ascorbic acid-Glutathione-Tocopherol cycle. Letters indicates the catalyzing enzymes in the individual reactions and red letters refers to those enzymes studied in this paper: A, tyrosine aminotransferase; B, HPPD, 4-hydoxyphenyl-pruvate dioxygenase; C, DXPR, 1-deoxy-D-xylulose-5-phosphate reductase; D, HGGT, homogentisate geranylgeranyl transferase; E, I, MPBQ-MT, 2-methyl-6-phytyl-1,4-benzoquinol methyltransferase; F, TC, tocopherol/tocotrienol cyclase; G, TMT, tocopherol/tocotrienol methyltransferase; H, HPT, homogentisic acid phytyltransferase; J, GLDH, L-galactono-1,4-lactone dehydrogenase; K, APX, ascorbate peroxidase; L, MDHAR, monodehydroascorbate reductase; M, DHAR, dehydroascorbate reductase; N, GR, glutathione reductase; O, GTPCH, GTP cyclohydrolase; P, DHN aldolase; Q, HPPK, 6-hydroxymethyldihydropterin pyrophosphokinase; R, DHPS, dihydropteroate synthase; S, DHFS, dihydrofolate synthase; T, DHFR, dihydrofolate reductase; U, FPGS, folylpolyglutamate synthase; V, ADCS, aminodeoxychorismate synthase; W, aminodeoxychorismate lyase; X, GMP, GDP-mannose pyrophosphorylase; Y, GME, GDP-mannose-3,5-epimerase; Z, VTC2, GDP-L-galactose phosphorylase. Abbreviations: HPP, 4-hydoxyphenyl-pyruvate; HGA, homogentisic acid; GGDP, geranylgeranyl diphosphate; DDXP, 1-deoxy-D-xylulose-5-p; GGMB, 6-geranylgeranyl-2-methylbenzene-1,4-diol; GGDMB, geranylgeranyl-2,3-dimethylbenzene-1,4-diol; PDP, phytyldiphosphate; MPQ, 1-methyl-6-phytyl-1,4-benzoquinol; DMPQ, 2,3-dimenthyl-5-phytyl-1,4-benzoquinol; MDHA, monodehydroascorbate; DHA, dehydroascorbate; GSH, glutathione; GSSG, oxidized glutathione; NADP, nicotinamide adenine dinucleotide phosphate; NADPH, nicotinamide adenine dinucleotide phosphate; GTP, guanosine triphosphate; DHN-P, dihydroneopterin phosphate; DHN, dihydroneopterin; HMDHP, 6-hydroxymethyldihydropterin; HMDHP-P_2_, 6-hydroxymethyldihydropterin diphosphate; DHP, dihydropteroate; DHF, dihydrofolate; THF, tetrahydrofolate; ADC, aminodeoxychorismate; pABA, p-aminobenzoate.

The biosynthetic pathways of vitamin C, E and folate (vitamin B_9_) in plants has been reviewed by a number of researchers^4–6^, their potential interdependent regulation is shown in Fig. 1. Vitamin C is a common compound existing in most living organisms and plays an important role in scavenging free radicals in living cells. The *L-galactose* pathway is the major pathway of vitamin C (*L-ascorbic acid*) biosynthesis in plants. This process begins with the phosphorylation of *D-Glucose* by hexokinase and ends with the epimerization of *L-Galactono-1*,*4-lactone* to *L-Ascorbic acid* by *L-galactono-1*,*4-lactone dehydrogenase*. Vitamin E consists of eight similar compounds: *α-tocopherol*, *α-tocotrienol*, *β-tocopherol*, *β-tocotrienol*, *γ-tocopherol*, *γ-tocotrienol*, *δ-tocopherol* and *δ-tocotrienol*. All of these compounds are lipid-soluble chemicals and are regarded as antioxidants. As Fig. 1 indicates, vitamin C and E work together in the cycle of ascorbic acid-glutathione-tocopherol, playing a role in eliminating free radicals on cell membrane^7^. The biosynthetic pathway of Vitamin E includes tocotrienol and tocopherol pathways. They both begin with *L-tyrosine* from shikimate pathway and end with series of methylation and cyclization. Folate, also called vitamin B_9_, is involved in DNA synthesis and thus it is critical for body development. Overall, the biosynthesis pathway of folate in plants is the assembly of a pterin, *a p-aminobenzoate* and polyglutamate tails in mitochondria^6^. Formation of *p-aminobenzoate* starts with *chorismate* which is one of the products of shikimate pathway, and ends in two steps in plastids^8^. Pterin synthesis firstly involves in the conversion of *guanosine triphosphate* to *dihydroneopterin triphosphate* via *guanosine triphosphate cyclohydrolase I* ^9^.

Sweet corn (*Zea mays* L.) produces kernels which are high in sugar content and also contain a range of bioactive ingredients. It is often consumed as a fresh or canned vegetable and is widely consumed globally^10^. Researchers have investigated the potential of germinating kernels, and using these as sprouts, to enhance the nutritional value of the material as well as providing a novel food product for consumers^11^. However, few reports evaluate the effect of germination on the nutritional quality of sweet corn, and the role of gene expression in manipulating germination and the production of nutrients. Hashim and Fields^12^ found that germinated kernels contain higher levels of riboflavin and niacin compared to the non-germinated kernels. Lay and Fields^13^ also stated that germinated kernels had significantly higher contents of ascorbic acid. Obizoba^14^ studied how the nutritional profile of normal yellow corn varied during germination and concluded that 24 or 36 hours of sprouting produced optimal levels of bioactive nutrients. Despite these research reports, there remains a paucity of information regarding the gene expression occurring during germination and how this is associated with changes of vitamins in different stages of sweet corn seedling. In addition, the presence or absence of light is considered as an important factor during seed sprouting. Researchers have reported that light germinated corn had higher phenolic contents as well as antioxidant activity compared to germination conducted in the dark^15^.

This study focuses on the change of vitamin C, E and folate as well as the relative expression of associated key genes in four major stages of sweet corn sprouting. Stage 1 representing initial radical emergence (typically after 48 hours), stage 2 representing chitting of the corn (typically after 72 hours), stage 3 representing emergence of the shoot and development of the root (typically after 96 hours), stage 4 representing shoot and root elongation (typically after 130 hours). Evaluations were conducted on samples sprouted in both light and dark environments. The intentions of this study were to: study the change of three selected kinds of vitamins during different periods of sweet corn spout; explore the molecular mechanism which were related to these changes; determine the effect of light and dark processing on nutrient composition in different stages of sweet corn sprouting; provide an insight of the complex biosynthetic pathways of selected vitamins.

## Results and Discussion

### Moisture contents in sweet corn sprouts during light and dark germination

**Figure 2:**
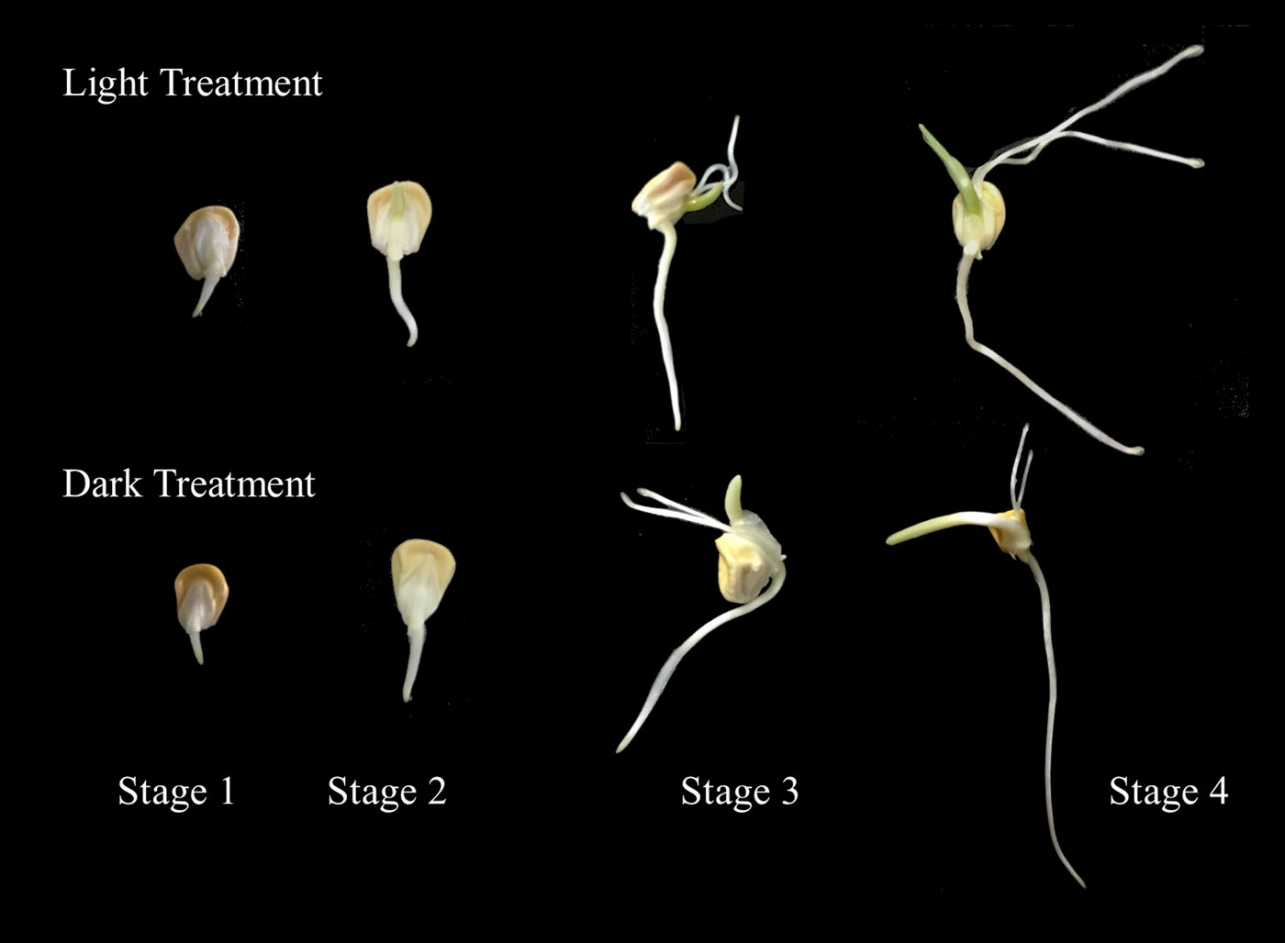
Sweet corn sprouts at different germination stages. Stage 1 representing initial radical emergence (typically after 48 hours); stage 2 representing chitting of the corn (typically after 72 hours); stage 3 representing emergence of the shoot and development of the root (typically after 96 hours); stage 4 representing shoot and root elongation with the appearance of euphylla (typically after 130 hours).

Moisture contents of germinating grains were determined according to the stages of sprout growth - For clarity, Fig. 2 illustrates the different stages of sprouting. The moisture contents of sweet corn sprouts in the four subsequent stages with light treatment were 53.51 ± 0.01%, 63.32 ± 0.02%, 63.80 ± 0.01% and 74.33 ± 0.01% respectively and those with dark treatment were 50.42 ± 0.02%, 58.41 ± 0.01%, 65.64 ± 0.02% and 70.27 ± 0.01% (n = 3). Moisture is required for seed germination^16^ and was used as a calibration factor in this study.

### Vitamin C and relevant key gene expression in sweet corn sprouts during light and dark germination

**Figure 3:**
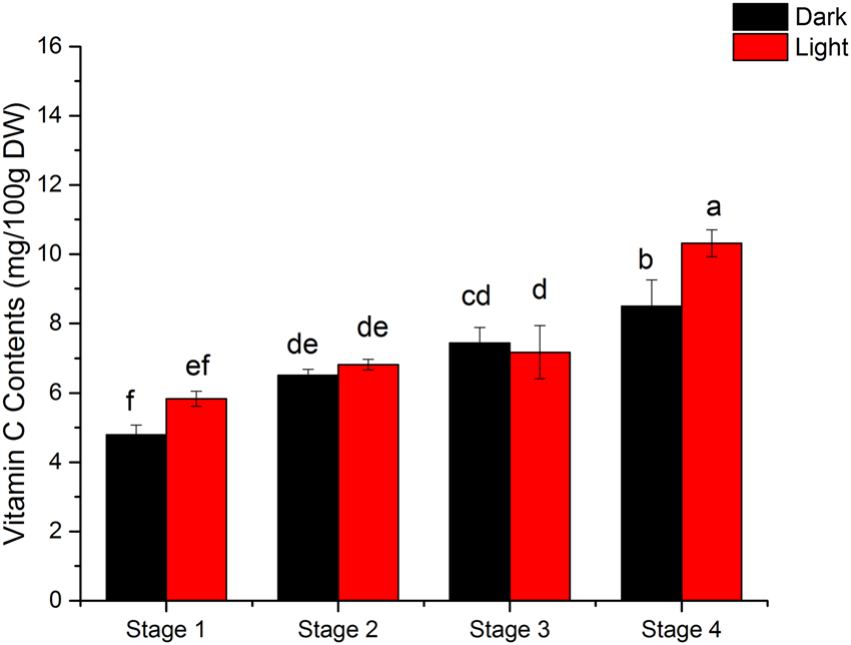
Changes of vitamin C during sweet corn seed germination; Bars with different letter indicates significant difference (p < 0.05) exists within groups.

**Figure 4:**
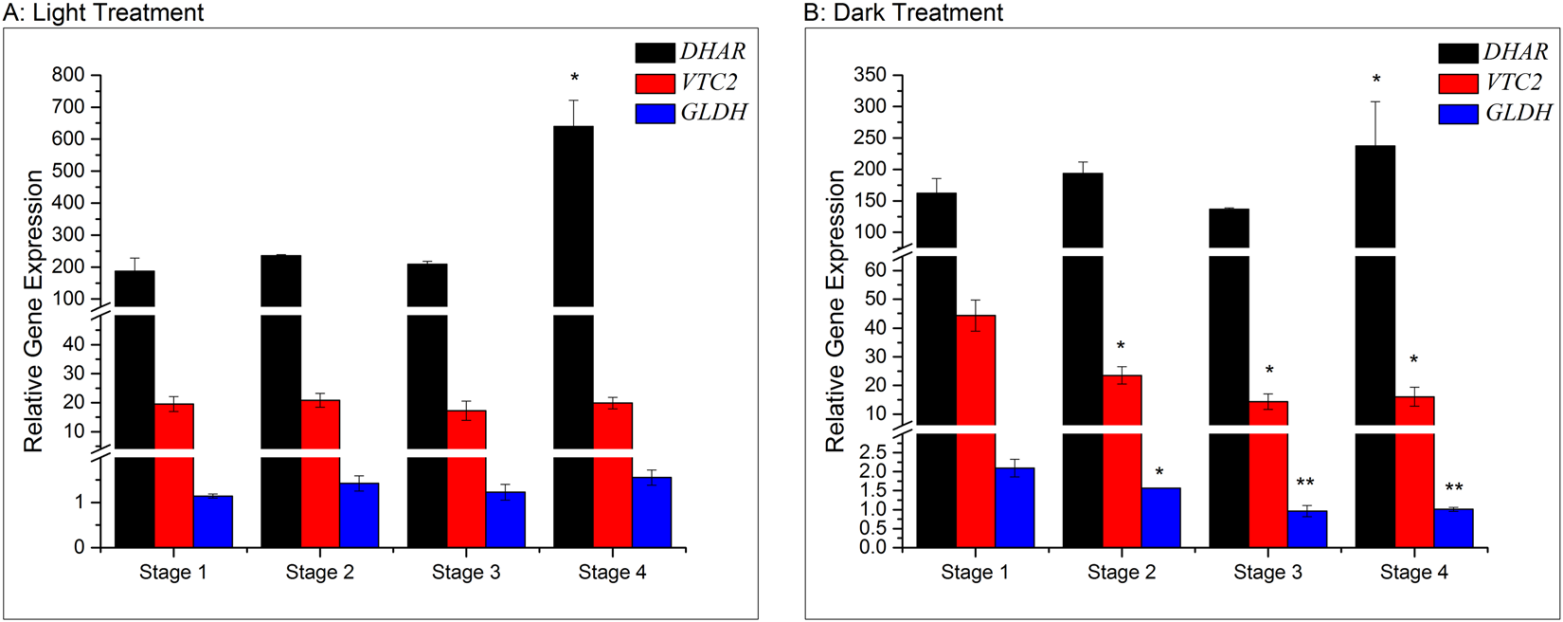
Changes of relative expression level of relevant genes in Vitamin C biosynthetic pathway during sweet corn seed germination; Bars with * and ** indicates significant difference at *p* < 0.05 and *p* < 0.01 respectively compared to stage 1. (**a**) Light treatment; (**b**) dark treatment.

Figure 3 demonstrates the changes of vitamin C. Overall, the contents of vitamin C increased during germination in both light and dark treatments. Three key genes (*VTC2*, *GLDH*, *DHAR*) affect the biosynthetic pathway of vitamin C^4, 17, 18^, the expression levels of these genes were studied and the results presented in Fig. 4. Compared to stage 1, the expression of *DHAR* gene in stage 4 of sprouting increased significantly (P < 0.05) from 186.9 ± 41.4 to 639.1 ± 81.8 and 162.3 ± 23.2 to 237.3 ± 70.4 under light and dark treatment respectively. The expressions of *VTC2* and *GLDH* were observed to be different. For instance, in the light group, the level of *VTC2* and *GLDH* mRNA had no significant difference during the sweet corn seed sprouting. However, the amounts of *VTC2* and *GLDH* mRNA declined significantly (*p* < 0.05) in the dark treatment from 44.34 ± 5.40 to 16.06 ± 3.28 and 2.09 ± 0.23 to 1.01 ± 0.05 respectively. The data shows that *DHAR* gene was much more actively expressed than *VTC2* and *GLDH* genes during sprouting.

Vitamin C works as an electron donor in animals and plants and is essential for repairing ROS (reactive oxygen species) damages in living cells^19^. Hence, vitamin C has become a standard to determine the nutritional quality of food. Our results illustrate that vitamin C levels increased during sprouting, specifically between the last two stages. Researchers have demonstrated previously that vitamin C was needed to scavenge the monolingual radicals during lignin biosynthesis in plant cells^20^. Therefore, as rapid growth of sweet corn sprouts occurred during the final two stages of sprouting, the rapid increase of vitamin C which was observed was reasonable. Similar phenomena have been recorded in soybean^21^ and mung bean^22, 23^ germination. For instance, Huang *et al*.^23^ reported that no vitamin C was found in non-germinated soybean and mung bean, whilst vitamin C increased during germination.

Previous research has indicated that vitamin C is generated by two ways: de novo synthesis from D-glucose, and the vitamin C recycling pathway called ascorbate-glutathione (*AsA-GSH*) cycle^24^. Three genes were selected in this study, two of which (*VTC2*, *GLDH*) were from *L-galactose* pathway and the other one (*DHAR*) was from the *AsA-GSH* cycle. It has been confirmed that *L-galactose* pathway is the main pathway to synthesize vitamin C in Arabidopsis^25^. *VTC2* and *GLDH* are involved in the transformation of *GDP-L-galactose* to *L-galactose-1-phosphate* and *L-galactono-1*,*4-lactoneto L-ascorbic acid* respectively (Fig. 1). The *AsA-GSH* cycle contributes to scavenging free radicals and regulating the vitamin C level in plants. Our results indicated that the AsA pool was extremely active during the sprouting of sweet corn seeds and the *AsA-GSH* cycle was the predominant way to regulate the vitamin C amount in sweet corn sprouts as the vitamin C contents in different germinal stages changed in relation to variations in the expression level of *DHAR* (Figs 3 and 4). *DHAR* is a critical enzyme for maintaining sufficient amounts of vitamin C in plant tissues^18^. Numerous studies have stated that plants with higher *DHAR* levels performed higher tolerance to extreme conditions^18, 26^. Wang *et al*.^27^ reported that the expression level of *DHAR* was the highest among the detected genes during soya bean germination. Sytykiewicz^17^ highlighted that *DHAR* accumulated during maize germination. Therefore, it can be hypothesized that the accumulation of vitamin C during sweet corn seedling was mainly attributed to the expression of *DHAR*.

The influence of light and dark treatment on vitamin C and relevant gene expression in sweet corn sprout was also studied. Previously Davey *et al*.^28^ reviewed the relationship between light, hexose and germination in detail. Significant differences existed in vitamin C contents as well as the gene expression. Figure 3 indicates that sweet corn seeds sprouted in the dark environment tended to have lower vitamin C level than in light environment. An increase of expression level of *VTC2* and *GLDH* during sweet corn germination occurred in the light environment while a significant (*p* < 0.05) decrease was observed in the dark environment (Fig. 4). D-glucose is the original material to vitamin C de novo synthesis, photosynthesis provides sufficient ingredients for the *L-galactose* pathway, causing higher level of expression of *VTC2* and *GLDH* and thus higher level of vitamin C. Gallie^18^ claimed that high light levels increased the production of ROS in plants. This observation might explain the higher expression level of *DHAR* in light germination, something that also agrees with previous research on the importance of *DHAR* to plant cells under different stress^17, 18, 20, 24, 26, 28, 29^.

### Vitamin E and relevant key gene expression in sweet corn sprouts during light and dark germination

Levels of *α-tocopherol*, *α-tocotrienol*, *β-tocopherol*, *γ-tocopherol*, *γ-tocotrienol*, *δ-tocopherol*, *δ-tocotrienol* as well as total vitamin E are presented in Table 1. Total vitamin E levels dropped significantly in the light samples from 9.08 ± 0.01 to 6.72 ± 0.38, 4.36 ± 0.03 and 4.31 ± 0.08 mg/100 g DW during the progressive stages of sprouting. A similar decrease was also observed in the dark environment. In light and dark groups, there was no significant difference of *β-tocopherol* content throughout sprouting, while the other six detected compounds declined during the germination stages with the highest levels in the initial stage of sprouting. *γ-tocotrienol* was the most abundant vitamin E compound in sweet corn seed sprouts, followed by *α-tocopherol*, *γ-tocopherol*, *α-tocotrienol*, *δ-tocotrienol*, *δ-tocopherol* and *β-tocopherol*. The amounts of *δ-tocopherol*, *α-tocopherol* and *γ-tocopherol* accounted for more than 80% of the total vitamin E in sweet corn sprouts. The contribution rate of *δ-tocotrienol* decreased during stage 1 and 2 of sprouting from 23% to 21% and 20% to 16% in light and dark treatment respectively, but increased between stage 3 and 4 from 18% to 26% and 16% to 20% in light and dark treatment respectively. The situation of *γ-tocotrienol* was opposite to that of *δ-tocotrienol* with its contribution rate increasing in the first three stages but decreasing in the final stage in both the light and dark groups.

**Table 1:** Change of Vitamin E profiles in sweet corn during germination.

Vitamin E Composition	Light treatment				Dark treatment			
	Stage1	Stage2	Stage 3	Stage 4	Stage 1	Stage 2	Stage 3	Stage 4
α-tocopherol	2.02 ± 0.25^a^	1.39 ± 0.15^b^	0.80 ± 0.02^c^	1.13 ± 0.07^b^	1.25 ± 0.14^b^	0.63 ± 0.04^c^	0.60 ± 0.02^c^	0.54 ± 0.03^c^
α-tocotrienol	0.76 ± 0.05^a^	0.67 ± 0.07^a^	0.47 ± 0.02^bc^	0.47 ± 0.03^bc^	0.51 ± 0.01^b^	0.38 ± 0.02^c^	0.39 ± 0.03^c^	0.29 ± 0.04^d^
β-tocopherol	0.14 ± 0.001^a^	0.16 ± 0.03^a^	0.12 ± 0.01^a^	0.13 ± 0.01^a^	0.10 ± 0.005^a^	0.09 ± 0.004^a^	0.10 ± 0.008^a^	0.09 ± 0.001^a^
γ-tocopherol	1.80 ± 0.19^a^	0.83 ± 0.57^b^	0.53 ± 0.06^b^	0.52 ± 0.05^b^	1.44 ± 0.09^a^	0.71 ± 0.06^b^	0.48 ± 0.03^b^	0.34 ± 0.005^b^
γ-tocotrienol	3.14 ± 0.20^a^	2.32 ± 0.47^b^	1.75 ± 0.16^bcd^	1.75 ± 0.21^bcd^	2.29 ± 0.06^bc^	1.60 ± 0.20^bcd^	1.35 ± 0.29^cd^	1.08 ± 0.12^d^
δ-tocopherol	0.24 ± 0.02^a^	0.22 ± 0.01^a^	0.16 ± 0.01^cd^	0.16 ± 0.01^cd^	0.21 ± 0.07^b^	0.16 ± 0.005^c^	0.18 ± 0.007^c^	0.14 ± 0.002^d^
δ-tocotrienol	0.55 ± 0.04^a^	0.49 ± 0.04^a^	0.40 ± 0.01^b^	0.38 ± 0.04^b^	0.39 ± 0.008^b^	0.35 ± 0.02^bc^	0.42 ± 0.02^b^	0.30 ± 0.01^c^
Total	9.08 ± 0.01^a^	6.72 ± 0.38^b^	4.36 ± 0.03^c^	4.31 ± 0.08^c^	6.20 ± 0.20^b^	3.92 ± 0.30^c^	3.67 ± 0.16^c^	2.78 ± 0.20^d^

Unit: mg/100 g DW; significance analysis was carried out within rows and data with different letters indicate significant difference at *p* < 0.05.

**Figure 5:**
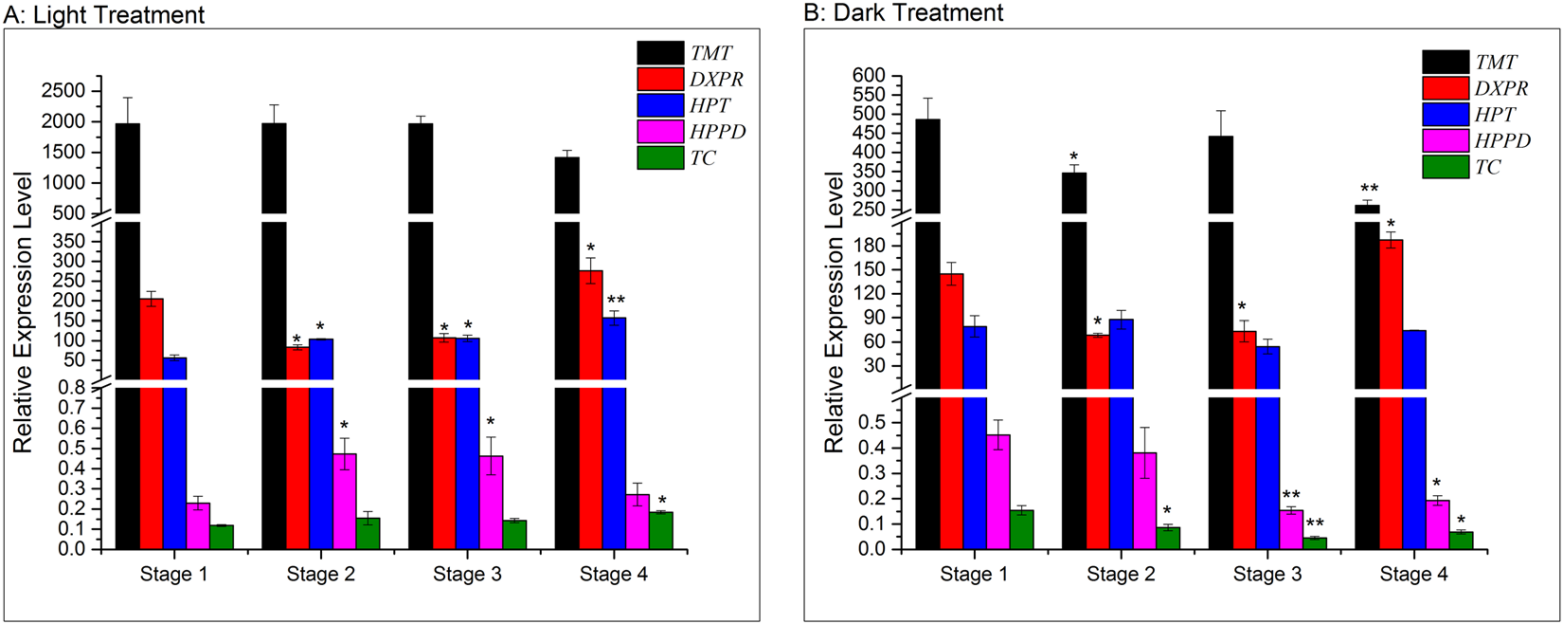
Changes of relative expression level of relevant genes in Vitamin E biosynthetic pathway during sweet corn seed germination; Bars with * and ** indicates significant difference at *p* < 0.05 and *p* < 0.01 respectively compared to stage 1. (**a**) Light treatment; (**b**) dark treatment.

To better understand the molecular mechanism behind these changes, five key-coding genes (*HPT*, *TC*, *TMT*, *DXPR* and *HPPD*) from the vitamin E biosynthetic pathway^30–32^ were selected for further investigation and their expression levels in sweet corn sprouts were evaluated (Fig. 5). *TMT* was the dominant gene among the five detected genes with the highest expression level. In light environment, the expression level of *TMT* was constant until stage 3 of sprouting and after that, it underwent a significant (*p* < 0.05) decrease from 1966 ± 127 to 1418 ± 118 between the last stages. The expression level of *TMT* in dark environment declined from 485.9 ± 56.2 to 346.4 ± 21.6 folds during stage 1 and 2, and then increased to 442.1 ± 66.9 folds at stage 3. *DXPR* was the second predominant gene in this study whose expression levels performed a typical U-curve during the sprouting stages in both light and dark groups. The expression level of *HPT* increased significantly (*p* < 0.05) during the light sprouting while showed little change during dark germination. *TC* and *HPPD* presented the lowest expression level in sweet corn sprouts. Overall, all the five selected genes tended to have higher expression levels in light seedling of sweet corn than the dark.

Vitamin E, including *tocopherols* and *tocotrienols*, are lipo-antioxidants in living organisms protecting cells against various oxidative stresses^30^. Sattler *et al*.^32^ reported that vitamin E was accumulated in seeds and critical for seed germination. However, few researchers have studied the vitamin E production during sweet corn sprouting. To our knowledge, most studies illustrate an increasing pattern of vitamin E contents during seed germination, including soya bean^27^, maize^33^ and sesame^34^. However, Kim *et al*.^35^ reported that decreased vitamin E level were observed in germinated rice. Bellani *et al*.^36^ showed different changing manners of tocopherols in different parts of radish seeds during germination with vitamin E contents in aged radish seeds decreased during a 24-hour-germination.

Our study showed a decreasing pattern of vitamin E in sweet corn sprouts. This phenomenon indicated that the consumption rate of vitamin E exceeded the production rate in sweet corn sprouts during germination causing rapidly decline of vitamin E levels. The reasons for this are currently unknown. We observed a significant (*p* < 0.05) increase of *α-tocopherol* in light conditions during the 2^nd^ and 3^rd^ stage of sprouting. This could be explained by the presence of green areas on the sprouts at these stages (*tocopherols* are synthesized by photosynthetic organisms)^32^. On the other hand, our study indicated that *γ-tocotrienol* was the main vitamin E in sweet corn sprouts. Researchers have confirmed that *tocotrienols* are the major isomers of vitamin E in different kinds of seeds, such as rice and wheat^30, 31^. The degree of seed dormancy might be another factor that influences the vitamin E composition in seeds. Numerous reports have documented the importance of vitamin E to seed quiescence and longevity^32^ as well as germination^37^. As a result, *tocotrienols* might play an important role in maintaining seed vigor during dormancy^38^.

*Homogentisic acid* (*HGA*) is the core compound in biosynthetic pathway of vitamin E. It can be involved in the biosynthesis of tocopherols as well as *tocotrienols* by reacting with *phytyldiphosphate* (*PDP*) and *geranylgeranyl diphosphate* (*GGDP*) respectively^39^. Many researchers have studied *4-hydoxyphenyl-pruvate dioxygenase* (*HPPD*) expression and illustrated an over-expression of *HPPD* may induce increases in vitamin E in barley, tobacco seeds and potato tubers^40^. Naqvi *et al*.^41^ found that synchronous expression of *HPPD* and *2-methyl-6-phytyl-1*,*4-benzoquinol methyltransferase* (*MPBQ*-*MT*) tripled the tocopherol level in corn kernels. *MPBQ*-*MT* is involved in downstream regulation of tocopherol biosynthesis.

We selected *HPT* as our focus as it is involved in the formation of *tocopherol*/*tocotrienol cyclase* (*TC*) and *tocopherol*/*tocotrienol methyltransferase* (*TMT*) are both needed in tocopherol and tocotrienol pathway triggering cyclization and methylation in downstream regulation respectively. Wang *et al*.^27^ reported the expression levels of *HPPD*, *MPBQ*-*MT*, *TC* and *TMT* exhibited irregular changes during germination but reached peak levels at 3 days after germination of soya beans, with *MPBQ*-*MT* being the predominant gene. However, the expression levels of genes in our study declined during germination. In addition, the results indicated that methylation was extremely active in sweet corn sprouts, because the expression level of *TMT* was much higher than other genes. Thus, we speculate that high levels of *γ-tocopherol*, *γ-tocotrienol*, *δ-tocopherol* or *δ-tocotrienol* was accumulated in sweet corn seed because these four isomers are methylated by TMT transforming to *α-tocopherol*, *α-tocotrienol*, *β-tocopherol* or *β-tocotrienol* respectively at the end of the vitamin E biosynthetic pathway. The speculation was based on the observations regarding the amounts of *γ-tocotrienol* being larger than any isomers in sweet corn sprouts. Although vitamin E and C are both antioxidants in living plant cells, they seemed to exhibit different metabolic mechanisms during sweet corn germination. Vitamin C could be mass synthesized during germination of sweet corn while vitamin E would be largely consumed which might be vast accumulated during sweet corn kernel maturation.

Exposure to a dark environment seemed to inhibit the expression level of the coding genes leading to smaller amounts of vitamin E in sweet corn sprouts. Several research have investigated the influence of light on vitamin E in plants^42, 43^ and shown that light could enhance the level of vitamin E as well gene expression possibly as a defense mechanism^44^. Therefore, the concentration of vitamin E as well as its relevant gene expression in sweet corn sprouts are light related.

### Folate and relevant key gene expression in sweet corn sprouts during light and dark germination

**Figure 6:**
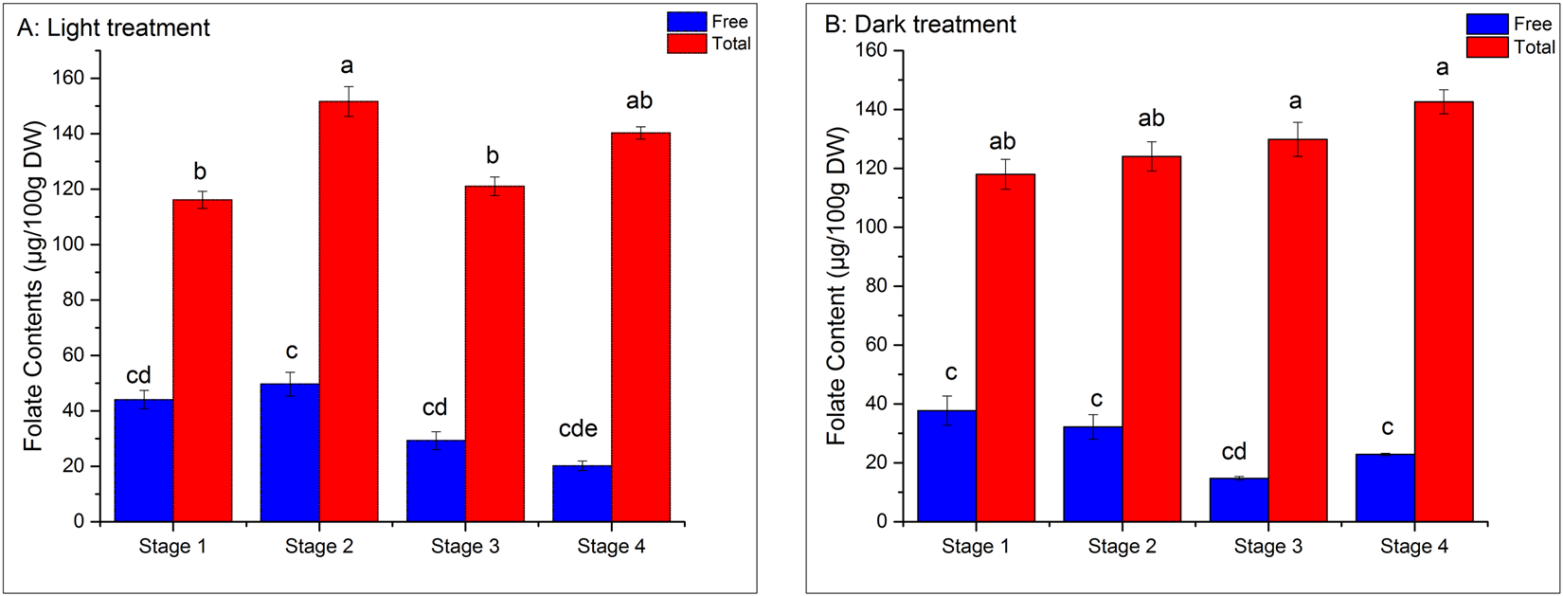
Changes of Folates during sweet corn seed germination; Bars with different letter indicates significant difference (*p* < 0.05) exists within groups. (**a**) Light treatment; (**b**) dark treatment. Free means readily extracted folate, which is obtained by free-enzyme extraction after autoclaving.

The effect of light and dark treatment on folate contents during sweet corn sprouting is presented in Fig. 6. In our studies, we characterized the folate content as free folate (the folate readily extracted without any enzymatic or thermal treatment) and bound folate (representing the difference between the folate recorded from extraction following thermal and enzymatic extraction – total folate content- and that which was readily extracted – free folate content). The free folate in light group increased during the first two stages of sprouting and then declined during the following stages of sprouting. The free folate in the dark group decreased until last stage of sprouting before increasing.

**Figure 7:**
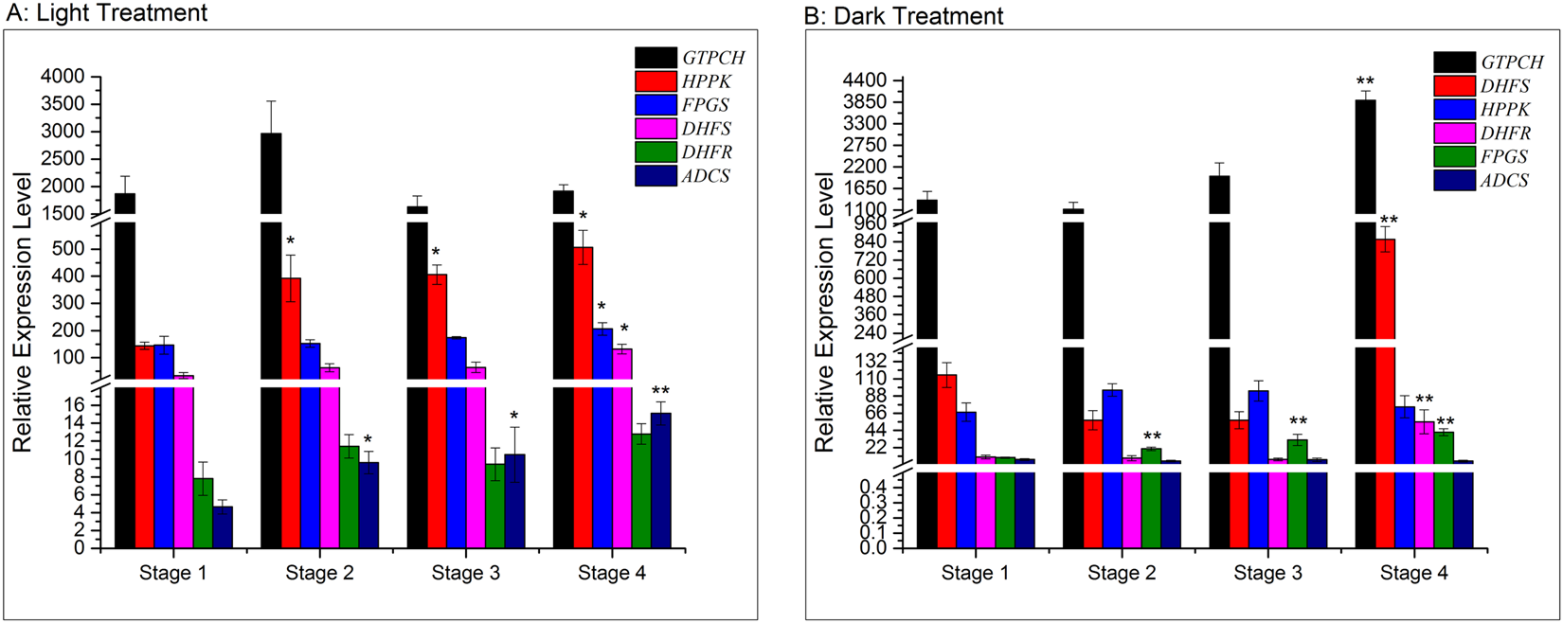
Changes of relative expression level of relevant genes in folate biosynthetic pathway during sweet corn seed germination; Bars with * and ** indicates significant difference at *p* < 0.05 and *p* < 0.01 respectively compared to stage 1. (**a**) Light treatment; (**b**) dark treatment.

The relative expression levels of six key-encoding genes of folate biosynthesis pathway^6, 45^ at different stages of sprouting with light and dark treatments are shown in Fig. 7. In the light group, *GTP cyclohydrolase* (*GTPCH*) was the most active gene among the six selected genes, but no significant change of expression level was observed during sprouting. *6-hydroxymethyldihydropterin pyrophosphokinase* (*HPPK*) was the second most abundant gene with expression levels increasing significantly (*p* < 0.05) during the first two stages of sprouting and then remaining unchanged from stage 2 to 4. The relative expression level of *folylpolyglutamate synthase* (*FPGS*) had no significant change until stage 3 after which it increased significantly (*p* < 0.05). *Dihydrofolate synthase* (*DHFS*) was inactive during the germination with no significant change of its relative expression levels. The relative expression level of *aminodeoxychorismate synthase* (*ADCS*) and *dihydrofolate reductase* (*DHFR*) increased significantly (*p* < 0.05) during sprouting. In the dark environment, *GTPCH* was also the most active gene among the six selected genes but its relative expression level decreased dramatically (*p* < 0.05) from stage onwards. Unlike the light group, *DHFS* was the second predominant gene and its relative expression level exhibited no significant change until the third stage of sprouting while it increased to the later stages. *HPPK* possessed a lower relative expression level amongst the other genes and was inactive during germination with the dark treatment. The relative expression level of *DHFR* remained unchanged during the former three stages and increased dramatically (*p* < 0.05) at stage 4. *FPGS* displayed a much lower relative expression level with light treatment, but its relative expression level increased significantly (*p* < 0.05) from stage 2 to 3 and remained unchanged at the next stages. *ADCS* was inactive during the germination with no significant change of its relative expression levels.

Folate is a general term for a series of compounds playing a critical role in transferring one-carbon unit^45^. Hefni and Witthöft^46^ found that germination resulted in a four-to-six-times increase in folate contents in wheat and rye seeds. Shohag *et al*.^47^ also reported two-to-four-fold increase occurred during legume seed germination. However, we found no articles reporting the situation in sweet corn seeds. In general, the total folate content increased in sweet corn seed sprouts during germination. Anukul *et al*.^48^ illustrated that *folate polyglutamylation* was required in the development of rice seeds. Folate are actively involved in the formation of amino acids as well as DNA; highly active during the germination^9, 49^. Folate have been found to be related with improving stress resistance of plants^50^. Interestingly, a peak was observed during the light germination at stage 2 but not in the dark group. Given the fact that light was the only variable in the test, it was reasonable to consider light as a regulating factor of folate biosynthesis during the sprouting of sweet corn seeds. Previously researchers have reported illumination enhanced total folate contents in lettuce^51^ and other crops. However, few researchers have studied the expression of relevant genes under light stimulus^52^.

It has been reported that folates are usually combined and bonded with different compounds forming food matrixes^53, 54^. Results indicated a reduction in free folate content of the sweet corn sprouts during germination. This suggests that the free folate gradually became conjugated in a compound matrix during the germination. In addition, we found that the contribution rate of free folate to the total amount of folate present in the sprouts declined over time from 38% to 33%, 24%, 14% in light environment and 32% to 26%, 11%, 16% in dark environment.

Six genes from different branches of folate biosynthetic pathway were chosen to understand the changing pattern of folate during sweet corn seed germination. *GTPCH* is involved in the formation of the pterin ring (Fig. 1) with researchers confirming that *GTPCH* was a rate-limiting enzyme manipulating the activity of folate pathway in *Arabidopsis thaliana* ^55^. It has been reported that *GTPCH* transcript could be found in various tissues of wheats^56^ and over-expression of *GTPCH* led to increase of folate contents in crops^57, 58^. Our results showed that *GTPCH* was the predominant gene among the six selected genes with the highest expression level. The levels of *GTPCH* gene relative expression in sweet corn sprouts during the germination in both light and dark groups was highly consistent with the variations observed in total folate content. This observation indicates that the transcript level of *GTPCH* may determine the amounts of total folate in sweet corn sprouts^56, 58^.

*ADCS* catalyzes the reaction of choirmate and glutamine providing the material for *p-aminobenzoate* (*pABA*) synthesis. This process takes place in plastids^45^ where photosynthesis also happen. Therefore, there might be some links between the activity of *ADCS* and light, as we observed that in light environment the relative expression level of *ADCS* increased, while in the dark the expression level of *ADCS* was lower. *HPPK*, usually combined with *dihydropteroate synthase* (*DHPS*), is involved in the production of *dihydropteroate* (*DHP*). Navarrete *et al*.^59^ investigated the role of *HPPK*/*DHPS* in *Arabidopsis thaliana* and found that besides taking part in folate synthesis, cytosolic *HPPK*/*DHPS* had a role in stress tolerance. Previous research has illustrated that illumination could induce various free radicals in plant cells^18, 28, 44^. Hence, the relative expression level of *HPPK* seemed to be light-related^60^ (Fig. 6). *DHFS*, *DHFR* and *FPGS* are encoding genes for synthesis of *dihydrofolate* (*DHF*), *tetrahydrofolate* (*THF*) and *polyglutamylated folate* respectively involved in downstream regulation of folate biosynthesis (Fig. 1). In general, all of their relative expression levels increased in different degrees, which could be explained by the requirement of nucleotide synthesis during sweet corn seed germination^60^. However, their relative expression levels had a significant (*p* < 0.05) increase with the dark treatments. Meng *et al*.^49^ suggested that *FPGS* was necessary for seed reserve accumulation and hypocotyl elongation in Arabidopsis under dark conditions. It is possible that the same phenomenon also occurred in sweet corn sprouts under dark environment. The extra function of *FPGS* compared to the light triggered the significant (*p* < 0.05) increase of the activity of *DHFS* and *DHFR* because of their interdependent relationship^6^.

### Potential interrelationships of the biosynthesis of vitamin C, E and folate in sweet corn sprouts

Indirect connections among vitamin metabolisms in plants exist and researchers have confirmed the relationship of N metabolism and folate biosynthesis in *atdfb-3* mutants^49^. Our study tried to provide an insight of the potential interdependent relationships among vitamin C, E and folate and the main breakthrough point would be their common role in biotic stress resistance in plants. A synergistic relationship between vitamin C and folate during the germination of sweet corn seeds was observed during this study, with both the levels of vitamin C and total folate increasing during germination. However, we also suggest that the active biosynthesis of folate may inhibit the activity of *L-galactose* pathway in sweet corn sprouts. GTP is needed in both pathways (Fig. 1). Therefore, high expression level of *GTPCH* and extremely low expression level of *GLDH* were detected in our experiment. Critical role of vitamin C in plant cell expansion and division, root growth and elongation have been reviewed thoroughly^28^. Gorelova *et al*.^45^ reviewed the metabolism of folate and illustrated accelerated total NADPH synthesis in animals and possibly also plants. Since NADPH is involved in ascorbate-glutathione cycle, which play an important part in ROS removal by reducing monodehydroascorbate into ascorbic acid. Folate accumulation in sweet corn sprouts might lead to accumulation of NADPH and thus contribute to the accumulation of ascorbic acid.

On the other hand, total vitamin E contents decreased over time with the increase of vitamin C in our study. This result contradicts the result from Wang *et al*.^27^, which reported the simultaneous increase of vitamin C and E in soya bean seed germination. Nevertheless, *α-tocopherol* can be regenerated from *α-chromanoxy* radical in ascorbate-glutathione cycle where vitamin C is oxidized to *MDHA* ^4^. Excessive vitamin C might generate extra *α-tocopherol* within cells, which would in turn trigger negative feedback to vitamin E biosynthesis. Therefore, vitamin C was supposed to compete or even inhibit the biosynthesis of vitamin E during the sweet corn seed germination.

In conclusion, the results revealed the changing pattern of vitamin C, E and folate during the germination of sweet corn sprouts in both light and dark environments as well as illustrating the molecular mechanisms behind these changes by studying the relevant key-encoding genes of these biosynthetic pathways. Further research is required to elaborate on these relationships. Such further study could involve the study of more genes as well as the determination of the levels of enzymatic pathways to disclose the activity of the whole metabolic pathways of different vitamins during sweet corn seed germination. In addition, genetic technologies, such as transgenic technology and gene knockout techniques could be applied to deeper study the interrelationship among different vitamins.

## Materials and Methods

### Sample preparation

Sweet corn seeds were obtained from crop research institute of Guangdong academy of agricultural science, Guangzhou, China). Intact seeds were selected by hand and then immersed in 75% alcohol for 1 min for sterilization. The seeds were soaked in distilled water for 2 hours. Next, the moistened seeds were divided into two groups and placed into glass containers with wet degreasing cotton and filter paper beneath. Each group was performed in triplicate. Both the light and dark groups were kept in an artificial climate chamber (PQX-330A-22A, Life Technology, Ningbo, Zhejiang, China) with temperature of 20 °C and illumination of 35000 LX. The dark group was wrapped with silver paper to avoid illumination. The cotton and filter paper were rewetted every 24 hours and the lamps were kept on during the experiment. Samples were collected at four different stages as shown in Fig. 2: Stage 1 representing initial radical emergence (typically after 48 hours), stage 2 representing chitting of the corn (typically after 72 hours), stage 3 representing emergence of the shoot and development of the root (typically after 96 hours), stage 4 representing shoot and root elongation (typically after 130 hours). About 50 seeds were used for germination in each container and about 6–7 seeds (typically 2 g) were collected for experiments. The collected samples were treated with liquid nitrogen and ground into flour. The samples were then stored at −80 °C until analysis.

### Measurement of moisture content

The moisture content was measured by the oven-drying method modified in our lab^21^. Samples (1 g) were placed in an oven at 105 °C until constant weight. The moisture of the sample was expressed as percent of dry weight (DW) in triplicate with data reported as mean ± SD (n = 3).

### RNA isolation, cDNA synthesis and quantitative real-time PCR analysis of relative gene expression

The extraction of RNA and subsequent cDNA conversion were carried out by Hipure Plant RNA Mini Kit (Megen, Guangzhou, China) and PrimeScript^TM^ RT Reagent Kit with gDNA Eraser (Takara Biotechnology, Dalian, China) according to the instruction booklets from the producers. Obtained RNA and cDNA were kept at −80 °C and −20 °C respectively until required for analysis. Real-time PCR was prepared using SYBP^®^ Premix Ex Taq^TM^ Kit (Takara Biotechnology, Dalian, China) and accomplished by LightCycler^®^ 480 Real-Time PCR System (F. Hoffmann-La Roche Ltd, Switzerland). The *ACTIN* (*GRMZM2G126010*) was selected as the reference gene^61^ and the primers used in this study were listed in Supplemental Table 1. PCR conditions were programmed as 95 °C for 3 min and 40 cycles of 95 °C for 5 s, 56 °C for 30 s, 72 °C for 30 s. Melt curve analysis was carried out following each real-time PCR operation. The efficiencies of the primers used were about 20. The concentration of mRNA was quantities before converting and so did cDNA before running RT-PCR. Cycle threshold (Ct) values were obtained after each operation which were around 17–22 and relative expression was calculated using the 2^−ΔΔCt^ method. Results are shown as mean ± SD (n = 3).

### Extraction and Determination of Vitamin C

Vitamin C content in sweet corn sprouts was extracted by 0.1% oxalic acid solution and determined by HPLC-PAD system (Waters Corp, Milford, MA, USA). About 0.2 g ground sample was dissolved in 0.1% oxalic acid solution and treated by Bioruptor^®^ sonication system (Diagenode Inc. USA) for 60 seconds. And then the mixture was brought to centrifugation. The upper liquid was collected and filtrated for the HPLC detection at the wave length of 245 nm. The HPLC conditions were: symmetry shield RP18 column (150 mm × 4.6 mm × 5 μm) with the temperature of 30 °C; methanol/oxalic acid (5:95, v/v) as affluent phase with the flow rate of 1.0 mL/min. Ascorbic acid was used as calibration. Data is expressed as milligram per 100 grams of dry weight sample (mean ± SD, n = 3).

### Extraction and Determination of Vitamin E

Extraction of vitamin E in sweet corn sprouts was conducted by the modified method described previously^62^. The determination of Vitamin E in sweet corn sprouts was carried out by a FL-HPLC system with a Waters 2475 Multi λ Fluorescence Detector at an excitation wavelength of 290 nm and an emission wavelength of 330 nm. The elution phase consisting of hexanes/isopropyl alcohol/acetic acid (99.05:0.85:0.1, v/v/v) with the flow rate of 1.0 mL/min was pumped by a Waters 515 HPLC Pump through a silica column (ZORBAX RX-SIL 4.6 × 250 mm, 5 μm, Agilent Technologies Inc., California, USA). The injection volume of extract samples was 20 μL. Identification of vitamin E was accomplished by the comparison of retention time between samples and standards. The quantification was carried out by standard curves. The outcomes were reported as milligram per 100 grams of dry weight sample (mean ± SD, n = 3).

### Extraction of Free and Total Folate

The extraction of folate was accomplished by the method used before^63^ and modified in our lab. For extraction of free folate, 0.5 g sample was mixed well with 10 mL disodium hydrogen phosphate in 15 mL centrifuge tube and then the mixture was sterilized by autoclaving the samples at 115 °C for 15 min. After cooling down, the solution was filtrated by vacuum filtration. The filtrate was collected and stored at −20 °C until determination. For extraction of total folate, 0.5 g sample was mixed well with 9.4 mL disodium hydrogen phosphate in 15 mL centrifuge tube and then the mixture was sterilized at 115 °C for 15 min. After the solution cooled down, 0.1 mL α-amylase (1.5 U/mg), 0.1 mL papain (≥6000 U/100 mg) and 0.4 mL chicken pancreas solution were added subsequently. After sufficient mix, the mixture was digested at 37 °C for 16 h with adequate shaking. After digestion, the tubes were placed in a boiling water for 3 min to inactive enzymes. Tubes were centrifuged and the total folate extracts were obtained by collecting the liquid through needle filter heads. Total folate extracts were kept at −20 °C until use.

### Determination of Folate Content

The determination of folate in sweet corn sprouts was carried out by standard assays^53, 54^. Briefly, each culture tube was prepared by 4.8 mL Milli-Q water, 5 mL Difco^TM^ Folic Acid Assay Medium (Sparks, MD, USA) and 0.2 mL folate extract. And then the tubes were sterilized at 121 °C for 20 min. After the tubes were cooled down, 50 μL *enterococcus hirae* (ATCC^®^ 8043^TM^, American Type Culture Collection, Manassas, VA, USA) suspension was added aseptically to each tube. Tubes were incubated at 37 °C for 19 h. When the incubation was finished, the tubes were brought to a 4 °C refrigerator for 15 min to stop the bacterial reproduction. Finally, the absorbance at 660 nm was read and recorded. The calculation was performed by a standard curve made by folic acid (Sigma, St. Louis, MO, USA). The results were reported as microgram of folic acid per 100 grams of dry weight sample (mean ± SD, n = 3).

### Statistics analysis

Statistics analysis was performed by OriginPro 2016 (OriginLab Corporation, Northampton, MA, USA). The differences among the sweet corn sprouts were determined by Tukey’s multiple comparison test (*p* < 0.05) with SPSS software 13.0 (SPSS Inc., Chicago, 1 L, USA).

## Electronic supplementary material

Supplemental Table 1
