# Supplementary material for: The manipulation of gene expression and the biosynthesis of Vitamin C, E and folate in light-and dark-germination of sweet corn seeds
Source: Sci Rep. 2017 Aug 8;7:7484. doi: 10.1038/s41598-017-07774-9 (PMC5548755; doi:10.1038/s41598-017-07774-9)
Supplement: Supplementary file 1 — Supplemental Table 1 [file 41598_2017_7774_MOESM1_ESM.doc]

**Title: The manipulation of gene expression and the biosynthesis of Vitamin C, E and folate in light-and dark-germination of sweet corn seeds.**

**Authors: Fengyuan Liu^1^, Nan Xiang^1^, Jian Guang Hu^2, 3^, Shijuan Yan^4^, Lihua Xie^1^, Charles Stephen Brennan^1, 5^, Wenjie Huang^4^, Xinbo Guo^1^, ***

**Affiliations:**

^1^School of Food Science and Engineering, South China University of Technology, Guangzhou510641, China.

^2^Crop Research Institute, Guangdong Academy of Agricultural Sciences, Guangzhou 510640, China

^3^Key Laboratory of Crops Genetics Improvement of Guangdong Province, Guangzhou, 510640, China

^4^Agro-Biological Gene Research Center, Guangdong Academy of Agricultural Sciences, Guangzhou 510640, China

^5^Department of Wine, Food and Molecular Bioscience, Lincoln University, Canterbury 7647, New Zealand

**Corresponding Author:**

* Xinbo Guo, E-mail: xbg720@gmail.com; Tel & Fax: (+86) 20-87113848

Supplemental Table 1. Primers used in qRT-PCR.

| Gene name | GeneID | Forward primer (5'-3') | Reverse primer (5'-3') |
| --- | --- | --- | --- |
| *VTC2* | 100272546 | ACCACAGTGCTATGCGGAGA | GGCCACTGAGCTCCCAGATT |
| *GLDH* | 103636542 | CACTGGGCCAAGATCGAGGT | GGTCTGCGCTAAGGAGTCCA |
| *DHAR* | 100285047 | GGTCTGCGCTAAGGAGTCCA | CCTCGAGCTTCACGATAGGCA |
| *DXPR* | 103634738 | GTGCTGGATCCAATGTCACG | TATGACACCTTGCTCCCCAG |
| *HPPD* | 606444 | ACTACTATGACGGCGTGAGG | CCTGGTCATCCCTATCCACC |
| *HPT* | 732789 | TCCATTGGCATCTGGGGAAT | TGCAGTCCCAAGAACAAAGC |
| *TC* | 541877 | GGGATGGAGAACGGTTTGA | CAGAAGCTCCTGGGAAGACA |
| *TMT* | 732837 | CCATCATCACCTGTCGCAAG | AGATGAGTAGACGGCGATGG |
| *GTPCHF* | 100193984 | GCGTTATGTGCAGTGGCTGA | GTGCAGCTCGGTTCGGATTG |
| *ADCS* | 103629593 | TAGCACGTGGCAGAACACCA | CATGAGGCGTGGGACATGC |
| *HPPK* | 100280079 | CAATGCCGCCGCTCTAACTC | CTAAGGCGCCTGTGGAACCT |
| *DHFS* | 103650558 | AGTGGTGTACCGGCGAGATG | GCCCTGCCTCAGTCAAATGC |
| *DHFR* | 541707 | AGGTATTTTGGCGTGGTGTT | ATCACCTTCCTCCCTGTGTG |
| *FPGS* | 100285702 | GCGGAATACGAGGAGGTGCT | TCCCAGCAACGTGAACCACT |
| *ACTIN* | 100282267 | TGTGGCTTTGGGATCGTAGTC | GAGCCACCGATCCAGACACT |
